# Molecular Detection of Integron Genes and Pattern of Antibiotic Resistance in Pseudomonas Aeruginosa Strains Isolated from Intensive Care Unit, Shahid Beheshti Hospital, North of Iran

**Published:** 2012

**Authors:** Fatemeh Moradian kouchaksaraei, Elaheh Ferdosi Shahandashti, Zahra Molana, Masomeh Moradian kouchaksaraei, Fariba Asgharpour, Ali Mojtahedi, Ramazan Rajabnia

**Affiliations:** 1 *Department of Microbiology Science and Research Branch Islamic Azad University, Guilan, Iran.*; 2 *Department of Microbiology and Immunology, Babol University of Medical Sciences, Babol Iran.*; 3 *Faculty of Para-Medicine; Babol University of Medical Sciences, Babol, Iran.*; 4 *Department of Microbiology Science and Research Branch Islamic Azad University, Fars, Iran.*; 5 *Infectious Diseases and Tropical Medicine Research Center, Babol University of Medical Sciences, Babol, Iran.*

**Keywords:** *Pseudomonas aeruginosa*, integrons, drug resistance

## Abstract

*Pseudomonas aeruginosa* is one of the most important pathogens that causes nosocomial infections and shows high level of antibiotic resistance. Integrons are one of the transposable elements in bacteria and their role in antibiotic resistance has been well demonstrated. The aim of this study was a molecular characterization of the integron genes and the determination of the resistance or sensitivity pattern to ceftizoxime, cephizoxim. cephotaxim, amikacin, ofloxacin, imipenem, cefepime, ticarcillin, gentamicin, ciprofloxacin, cefazolin and ceftriaxone antibiotics in *P. aeruginosa* strains isolated from Intensive Care Units (ICU), Shahid Beheshti Hospital, North of Iran. This cross-sectional study was performed from 2011 to 2012. Totally, fifty four *P. aeruginosa* strains were isolated from ICU at Shahid-Beheshti hospital, Babol, North of Iran. The bacteria were diagnosed based on mobility, pigment production, growth in 42^0^ C, oxidase and catalase tests. PCR analysis was carried out to detect integron genes using hep 35 and hep 36 primers. Also, disk diffusion method was performed to evaluate antibiotic susceptibility of the bacteria using ceftizoxime, ceftazidime, cephotaxime, amikacin, ofloxacin, imipenem, cefepime, ticarcillin, gentamicin, ciprofloxacin, cefazolin and ceftriaxone antibacterial reagents. This study revealed that 20 (37%) *P. aeruginosa* isolates had integron genes. The antibiotic susceptibility test showed that 53 (98.1%) of the isolates were multidrug-resistant. 12 out of 54 isolated bacteria were resistant to all antibiotics tested. All bacteria were resistant to cefepime and the highest resistance rate was seen to ceftizoxime 92.6% followed by cefazolin 92.3%. The lowest resistance rate was observed to ciprofloxacin 38.9%, ofloxacin 44.4%, amikacin 46.3% and ticarcillin 48.1%. According to this study, *P. aeruginosa* isolates showed high level of antibiotic resistance and the presence of integrons in these strains can explain the influence of these genes in resistance creation. There was a significant association between resistance to cefotaxime, amikacin, ofloxacin, imipenem, ticarcillin, gentamicin and the presence of integrons.

*Pseudomonas aeruginosa* is a non-fermenting aerobic Gram negative microorganism known as a pathogen leading to severe infections in hospitals. This organism is naturally resistant to many antimicrobial agents and it also can acquire the resistance against available antibiotics through multiple mechanisms (1). In the recent years due to the resistance of these pathogenic bacteria to different antibiotics, treatment of their infections has encountered a number of difficulties and many patients died because of these types of infections. The resistance mechanisms of these bacteria can be explained by either the mutation of genes or vertical transmissions through transformation or conjugation. Therefore the antibiotic resistance genes can be transported by plasmids and transposons between cells (2, 3). 

Lately a new group of transportable genetic elements has been identified which can transfer the antibiotic resistance genes. This genetic element has been called integron and can surround genes and transfer them while they are in the gene cassettes. Integrons are classified into four classes according to their integrase genes, and the class 1 integron is the prevalent one (4, 5, 6). 

Class 1 integron is composed of a 5'-conserved segment (5'CS) including the intI and attI genes and Pant promoter and 3'-conserved segment (3'CS) encoding resistance to sulfonamides (SulI) and disinfectants (7, 8). Class 2 integron is similar to class 1 and it contains integrase gene (intI2), one recombinant location (aatI 2) and a cassette gene without SulI in its 3' terminal region. The class 2 integron is less abundant than class 1 integron among Gram negative bacteria with antimicrobial resistance (9). Integron class 3 has intI3 gene, attI3 site and promoter and it is structurally identical to class 1 integron (10). Due to an important role of Integrons in antimicrobial resistance, a wide range of studies has been done on this gene. In Iran, an experiment has been performed in Imam Hospital of Uromieh that studied the influence of class 1 integron on the resistance of *P. aeruginosa* during 2007 to 2008 (11). Also the presence of class 1 and 2 integrons was studied in south of China during 2001 to 2005 (1).

In our previous study we investigated class 1 integron in *Pseudomonas aeruginosa* isolates from different places and devices of ICU in Shahid Beheshti Hospital during 2008 to 2009 (12). The aim of the present study was to perform a molecular analysis of all classes of integrons with hep primers in *Pseudomonas aeruginosa* strains isolated during 2011-2012 from the intensive care unit (ICU), and to determine their antibiotic resistance pattern as well as comparing these recent findings with those reported in our previous study.

## Materials and Methods

**Bacterial strains**

This cross - sectional study was performed on 54 *P. aeruginosa* isolated from equipment and devices of ICU of Shahid - Beheshti hospital of Babol during 2011-2012. After collection, samples were cultured in EMB and blood agar media (Merck.Germany) and incubated at 37^0^C for 24 hours. The gram negative bacilli were analyzed using differential tests in order to identify the* P. aeruginosa* strains. These tests include: mobility, pigment production, growth at 42^0^ C, oxidase and catalase tests.

**Antimicrobial susceptibility testing**

The susceptibility of the *P. aeruginosa* strains to cephotaxime (CTX), amikacin (AN), ofloxacin (OFX), imipenem (IPM), cefepime (FEP), ticarcillin (TIC), gentamicin (GM), ceftazidime, (CAZ), ciprofloxacin (CP), cefazolin (CZ), ceftriaxone (CRO) and ceftizoxime (CT) antibiotics was determined by disc diffusion method (Padtan Teb Co, Iran) and the data were analyzed according to clinical and laboratory standards institute (CLSI 2006).

**DNA extraction and polymerase chain reaction**

DNA of isolates was extracted using High pure PCR Template preparation Kit (Roche Co, Germany). The PCR was performed in 50 µl volumes that contained 10 µl of extracted DNA, 50 pMole of each primer (Copenhagen, Denmark), 30 µl distilled water, 5 µl of 10X buffer, 1.5mMole MgCl2, 0.2 mMole dNTPs and 1.5 U of Taq DNA polymerase enzyme (Copenhagen, Denmark). The presence of integrons was detected by hep primers in each isolate but this primer does not determine the class of integron. The hep 35 with the sequence of 5’ TGCGGGTYAARGATBTKGATTT 3’ and hep 36 with the sequence of 5’ CARCACATGCG-TRTARAT 3’ were used for amplification of integrons gene (13).

Amplification of a 491bp fragment was conducted in a thermocycler as follows: a primary denaturation at 94^0^C for 5 minutes followed by 30 cycles of denaturation at 94^0^C for 30s, annealing at 55^0^C for 30s and extension at 72^0^C for 45s and one cycle of final extension at 72^0^C for 10 min. After performing PCR reaction, electrophoresis of PCR product was conducted in 1.5% agarose gel (Cinagene Co, Iran) and the results were evaluated in the presence of 100 bp DNA size marker (Fermentas Co, Ukraine). 

**Statistical analysis**

The data were analyzed using the SPSS software version 18. The Chi-square test was employed to calculate the P value in terms of resistant, intermediate and susceptible numbers of integron-positive and negative isolates.

## Results

**Fig 1:**
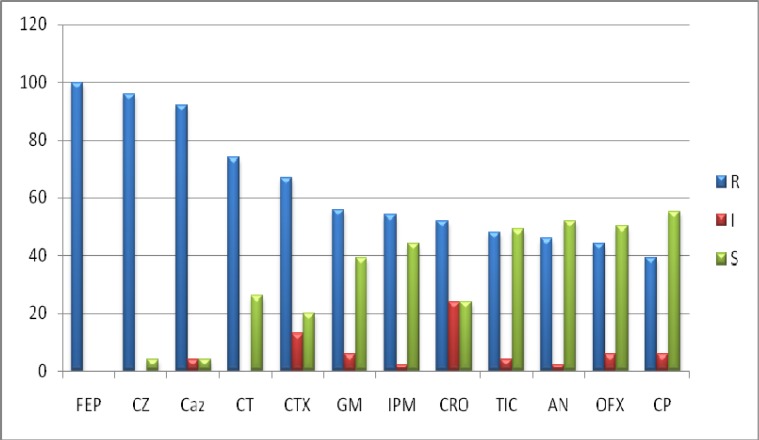
**. **The antibiotic resistance and susceptibility of *P. aeruginosa* strains isolated from Shahid Beheshti hospital ICU of Babol to different antibiotics

Fifthy four isolated* P. aeruginosa* strains from Shahid Beheshti hospital ICU of Babol were tested for their resistance or susceptibility pattern to ceftizoxime, ceftazidime, cephotaxime, amikacin, ofloxacin, imipenem, cefepime, ticarcillin, gentamicin, ciprofloxacin, cefazolin and ceftriaxone antibiotics by disc diffusion method. The highest resistances were against cefepime 100%, ceftazidime 97% and cefazolin 92.3% and the lowest were against ciprofloxacin 38.9%, ofloxacin 42.5%, amikacin 46.3% and ticarcillin 48.1% (Fig.1). 

Furthermore, multi-drug resistant *P. aeruginosa* strains were studied. Twelve strains of 54 isolated* P. aeruginosa* were resistant to all antibiotics. Also 52 strains (96.3%) had shown resistance to 3 or more antibiotics and the second frequent group of this study was resistance to CT, CTX, CZ, CAZ, and FEP. In addition, the lowest frequency distribution of multi-drug resistance was seen in one isolate with resistance to two antibiotics Table 1; and only one isolate was susceptible to all antibiotics.

**Table 1:** multi- drug resistance in *P. aeruginosa *strains isolated from Shahid Beheshti hospital ICU of Babol by disc diffusion method

**Total number** **of resistant** **isolates (%)**	**Antibiotics No.**	**Number** **of antibiotic** **resistant isolates**	**Antibiotics**
12 (22.2%)	12	12	FEP-CAZ-CZ-CT-CTX-CRO-AN-GM-TIC-IPM-OFX-CIP
10 (18.5%)	11	1 2 2 3 1 1	FEP-CAZ-CT-CTX-CRO-AN-CZ-IPM-TIC-CIP-OFX FEP-CAZ-CT-CTX-CRO-GM-TIC-CZ-AN-IPM-OFX FEP-CAZ-CT-CTX-CRO-CZ-OFX-GM-TIC-CIP-IPM FEP-CAZ-CZ-CT-CTX-IPM-GM-CIP-TIC-CRO-AN FEP-CZ-CAZ-CT-CTX-IPM-GM-CIP-CRO-AN-OFX FEP-CZ-CAZ-CTX-IPM-GM-CIP-CRO-AN-OFX-TIC
2 (3.7%)	10	2	FEP-CAZ-CTX-CRO-GM-TIC-IPM-CZ-CT-AN-
1 (1.9%)	9	1	FEP-CAZ-CT-CTX-CRO-TIC-GM–CZ-TIC-
3 (5.5%)	7	1 1 1	FEP-CZ-GM-CAZ-CRO-OFX-IPM FEP-CZ-AN-GM-CAZ-OFX-CT- FEP-CZ-CAZ-CRO-OFX-IPM-CT
2 (3.7%)	6	1 1	FEP-CZ-CAZ-CRO-CTX-CT FEP-CZ-AN-GM-CZ-CTX
9 (18.5%)	5	1 1 1 6	FEP-CAZ-CZ-GM-AN FEP-TIC-CRO-CTX-CAZ FEP-CZ-OFX-CTX–IPM FEP-CAZ–CZ-CTX–CT
6 (11.2%)	4	4 1 1	FEP-CZ-CAZ-CT FEP–CZ-CAZ-CTX FEP-CZ-AN-CT
7 (12.9%)	3	1 5 1	FEP-CAZ-TIC- FEP–CAZ-CZ FEP-CTX-CZ
1 (1.9%)	2	1	FEP-CZ

**Fig .2:**
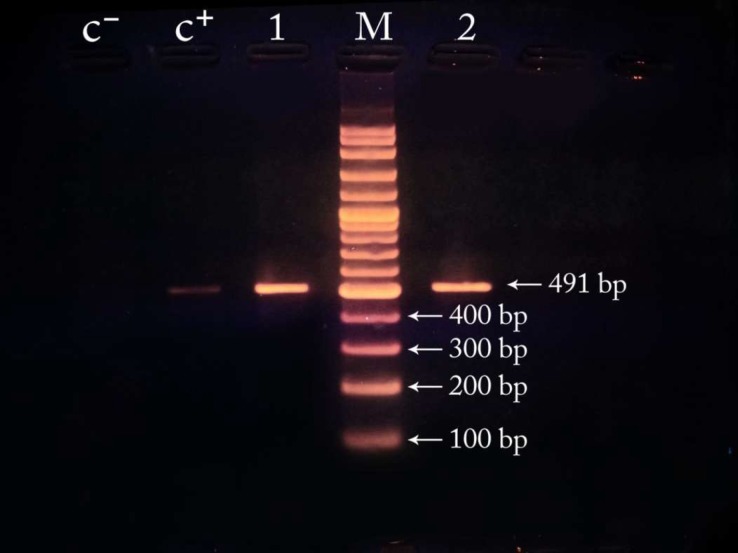
Gel-Electrophoresis of the PCR products of integrons by hep primers (I lanes) and control samples (positive control: C+, negative control: C-).

PCR analysis, using hep35 and hep36 primers, amplified an expected band on agrose gel (491 bp) in 20 out of 54 isolates of *P. aeruginosa* and therefore 37% of the studied isolates had the integron genes (Fig. 2). Furthermore, all the integron positive strains were multi-drug resistant, and among them nine (45%) and four (20%) isolates were respectively resistant to twelve and eleven antibiotics which used in the current study. (Table 2). Table 2 shows antibiotic susceptibility of integron-positive and integron-negative strains of *P. aeruginosa.* 57% of isolates resistant to Ceftriaxone, 62% of isolates resistant to Ciprofloxacin, 59% of isolates resistant to Imipenem, 56% of isolates resistant to Amikacin and 57% of isolates resistant to Gentamicin had integron genes (Table 2).

**Table 2:** Antibiotic susceptibility of Integron-Positive and Integron-Negative strains of *P. aeruginosa *isolated from

**Antibiotics**	**disc diffusion** ** method** **n=54**			**Integron Positive** n=20			**Integron Neg**ati**ve** n=34			**P value**
	R % (n)	I % (n)	S % (n)	R	I % (n)	S	R % (n)	I	S	
				% (n)		% (n)		% (n)	% (n)	
imipenem	53.7% (29)	1.9% (1)	44.4% (24)	85% (17)	0% (0)	15% (3)	35.3% (12)	2/9% (1)	61/8% (21)	0.01
cefepime	100% (54)	0% (0)	0% (0)	100% (20)	0% (0)	0% (0)	100% (34)	0% (0)	0% (0)	NS
ticarcillin	48.1% (26)	3.8% (2)	48.1% (26)	80% (16)	0% (0)	20% (4)	29.4% (10)	5/9% (2)	64/7% (22)	0.00
ofloxacin	44.4% (24)	5.6% (3)	50% (27)	75% (15)	0% (0)	25 (5)	25% (9)	9/4% (3)	65.6% (22)	0.01
amikacin	46.2% (25)	1.9% (1)!	51.9% (28)	70% (14)	5% (1)	25% (5)	32.4% (11)	0% (0)	67.6% (23)	0.04
gentamicin	55.5% (30)	5.6% (3)	38.9% (21)	85%. (17)	0% (0)	15% (3)	38.2% (13)	8.8% (3)	53% (18)	0.02
cefazolin	96.3% (52)	0% (0)	3.7% (2)	100% (20)	0% (0)	0% (0)	94.1% (32)	0% (0)	5.9% (2)	NS
ceftriaxone	51.8% (28)	24.1% (13)	24.1% (13)	80% (16)	10% (2)	10% (2)	35.2% (12)	32.4% (11)	32.4% (11)	0.07
ceftizoxime	74.1% (40)	0% (0)	25.9% (14)	90% (18)	0% (0)	10% (2)	64.7% (22)	0% (0)	35/3% (12)	0.05
cefotaxime	66.6% (36)	13% (7)	20.4% (11)	85% (17)	0% (0)	15% (3)	55.9% (19)	20/6% (7)	23.5% (8)	0.04
ciprofloxacine	38.9% (21)	5.6% (3)	55.5% (30)	65% (13)	0% (0)	35% (7)	23.5% (8)	8.8% (3)	67.7% (23)	0.05
Ceftazidime	92.6% (50)	3.7% (2)	3.7% (2)	100% (20)	0% (0)	0% (0)	88.2% (30)	5/9% (2)	5/9% (2)	NS

R: resistant; I: intermediate; S: sensitive

## Discussion

In this study the presence of integron genes in *P. aeruginosa* strains was investigated and antimicrobial susceptibility test was performed. A high proportion of isolated strains showed high resistance to investigated antibiotics. The presence of integron genes among resistant* P. aeruginosa* isolates confirms the role of this gene in resistance of strains and validates the origin of these bacteria from one common colony. Hence, there is a high risk of infection in hospitalized patients of ICU and due to increase of antibiotic resistance in isolated strains; their treatment is becoming a matter of concern. Therefore some strategies should be employed to inhibit the increasing resistance of these strains and the spread of resistance genes between strains and prevent the colonization of these bacteria in hospital and especially in its ICU.

In our previous study we investigated class 1 integron in *Pseudomonas aeruginosa* isolates from ICU during 2008 to 2009 (12) and found that 39.4% of isolates had intl gene among which 24.2% were multidrug-resistant. While in this study 96.3% were multidrug-resistant. This difference could be due to the increased resistance or difference in methods used in susceptibility testing.

In this investigation the rate of susceptibility to ceftizoxime, ceftriaxone, ciprofloxacin, ofloxa-cin, was 25.9%, 24.1%, 55.5%, and 50%, respectively. These data are quite in line with Fuladi et al. results that reported 23% susceptibility to ceftizoxime and 29% to ceftriaxone but they found 36% susceptibility to ciprofloxacine and 70% to ofloxacin (14). In addition, 98.1 % of isolated strains showed multi-drug resistance (MDR) which is higher than 87.05% of MDR reported in Mirsalehian et al.'s study (15). 

Here, the resistance of isolated *P. aeruginosa* to ticarcillin, cefazolin, cephotaxim, ceftriaxone, cefepime, amikacin, gentamicin, ciprofloxacin antibiotics were 48.1%, 96.3%, 66.7%, 51.9%, 100%, 46.3%, 55.6% and 38.9% respectively, while Chang and his colleagues in 2009 studied *klebsiella pneumoniae*, another cause of infection in hospital, and reported 9.2%, 21%, 3.7%, 5.8%, 0.3%, 9.2%, 17.4% and 14.8% rate of resistance to the same antibiotics (16). These data disclose the high rate of resistance and decreased rate of susceptibility of strains to different antibiotics in Iran which can be owing to hygienic statement, epidemiology of the region and different strains of bacteria in the studied region (14).

Also in a study that has been performed in United States during 2001 to 2003, most of multi-drug resistant strains (29.7%) were resistant to 4 antibiotics including ceftazidime, imipenem, gentamicin, ciprofloxacin or ofloxacin while in our investigation 22.2% of isolated strains were resistant to 12 antibiotics and 11.1% of specimens showed resistance to cefepime, ceftazidime, cefazolin, cephotaxim, ceftizoxime. These data reveal the increasing rate of antibiotics resistance in Iran and all over the world (17).

Several studies in Iran have disclosed the critical condition of multi-drug resistance strains especially* P. aeruginosa* in hospital infections (18). Accordingly, studying the resistance pattern of these bacteria is an important subject. Increasing the resistance of* P. aeruginosa* to multiple antibiotics cause multiple drug regimens recruitment for those infections.

Several factors are involved in multi-drug resistance of *P. aeruginosa* among which change of membrane’s permeability, plasmids and integrons are important (19). The presence of integrons was detected by hep primers in each isolate, but this primer does not determine the class of integrons. The frequency of integrons in this study was 37% while Ruiz Martinez and his colleagues in Spain and Bing Gu et al. in China reported respectively 34.2% and 40.7% integrons frequency (20, 21).

In our investigation, 18 isolates (33.3%) were resistant to imipenem, ceftazidime, cefepime, ciprofloxacin and amikacin antibiotics among which, 14 (70%) had integrons; While a clinical study in Turkey on 67 isolates of *P. aeruginosa* from ICU showed that 79.1% were sensitive to imipenem, ceftazidime, cefepime, ciprofloxacin and amikacin antibiotics and only 14 (20.8%) were resistant to all putative antibiotics and in this group only two strains had integron (22). The different pattern of antibiotics resistance in Iran in comparison with other countries can be explained in part by its geographical diversity, the place of strains isolation and antibiotic usage.

In this research, the resistance of integron positive strains to gentamicin, ticarcillin, ciprofloxacin, imipenem, amikacin, ceftriaxone, ofloxacin, ceftazidime and cefepime antibiotics was 81.9%, 77.3%, 54.5%, 81.1%, 63.6%, 77.3%, 72.7%, 100% and 100% respectively. These data showed that the presence of integron is significantly associated with resistance to gentamicin, ticarcillin, imipenem, amikacin, cefotaxime and ofloxacin antibiotics. Moreover, all of the integron positive strains were resistant to cefepime and ceftazidime but there was no significant association between the presence of integrons and the resistance to these two antibiotics.

This investigation can be compared with the study of Jianguo Chen and his colleagues in China where 38% of 71 isolated strains had integron and 88.9% of integron positive specimens were resistant to ceftriaxone, 92.6% to gentamicin, 48.1% to ceftazidime, 59.3% to ciprofloxacin, 25.9% to imipenem, 63% to cefepime, 66.7% to amikacin and 92.6% to cephotaxim (23). These results indicate that the presence of integrons can be effective in antibiotic resistance of *Pseudomonas aeruginosa *cases.

In our study, all 20 integron positive specimens showed multi-drug resistance and this may confirm the role of integrons in resistance of these bacteria to different antibiotics. 

Since different groups of antibiotics have diverse effect on *Pseudomonas*, other mechanisms of resistance may be involved in this process (24, 25). In our study, we did not find any specimen sensitive to all antibiotics. Decreasing the rate of sensitivity of these bacteria to multiple antibiotics can lead to many difficulties concerning the treatment of such infections. 

Our data revealed that high rate of resistance of *Pseudomonas* to cefepime, ceftazidime, ticarcillin, imipenem, ofloxacin, cefazolin and ceftriaxone makes these antibiotics as an inappropriate drug for treatment of *Pseudomonas*’ infections. It is possible that in the near future with the expansion of multudrug resistance, ciprofloxacin and amikacin antibiotics lose their influence on *Pseudomonas*. In our investigation we observed some specimens that were resistant to different antibiotics inspite being integron negative. Therefore we should also look for other factors that make these bacteria resistant to antibiotics. 
